# Remaining Useful Life Prediction from 3D Scan Data with Genetically Optimized Convolutional Neural Networks

**DOI:** 10.3390/s21206772

**Published:** 2021-10-12

**Authors:** Giovanni Diraco, Pietro Siciliano, Alessandro Leone

**Affiliations:** IMM—Institute for Microelectronics and Microsystems, National Research Council of Italy, 73100 Lecce, Italy; pietro.siciliano@le.imm.cnr.it (P.S.); alessandro.leone@cnr.it (A.L.)

**Keywords:** remaining useful life, deep neural network, convolutional neural network, genetic optimization, neural network optimization, support vector regression, depth maps, normal maps, 3D point clouds

## Abstract

In the current industrial landscape, increasingly pervaded by technological innovations, the adoption of optimized strategies for asset management is becoming a critical key success factor. Among the various strategies available, the “Prognostics and Health Management” strategy is able to support maintenance management decisions more accurately, through continuous monitoring of equipment health and “Remaining Useful Life” forecasting. In the present study, convolutional neural network-based deep neural network techniques are investigated for the remaining useful life prediction of a punch tool, whose degradation is caused by working surface deformations during the machining process. Surface deformation is determined using a 3D scanning sensor capable of returning point clouds with micrometric accuracy during the operation of the punching machine, avoiding both downtime and human intervention. The 3D point clouds thus obtained are transformed into bidimensional image-type maps, i.e., maps of depths and normal vectors, to fully exploit the potential of convolutional neural networks for extracting features. Such maps are then processed by comparing 15 genetically optimized architectures with the transfer learning of 19 pretrained models, using a classic machine learning approach, i.e., support vector regression, as a benchmark. The achieved results clearly show that, in this specific case, optimized architectures provide performance far superior (MAPE = 0.058) to that of transfer learning, which, instead, remains at a lower or slightly higher level (MAPE = 0.416) than support vector regression (MAPE = 0.857).

## 1. Introduction

Over recent years, asset maintenance has received increasing attention in the literature. If considering that appropriate maintenance management has a direct effect on reducing costs and increasing system reliability, availability, and safety [1], it is easy to understand how asset maintenance is a critical success factor in the current industrial revolution 4.0 [2]. More specifically, the adoption of optimized maintenance strategies has been proven to improve tool utilization and productivity, assuring product quality and operational excellence [3].

With the main objective of reducing unexpected breakdowns and possibly catastrophic consequences, maintenance strategies can be roughly classified under [4]: (1) corrective maintenance, (2) preventive maintenance, and (3) prognostics and health management (PHM). In the case of corrective maintenance, machine tools are operated until the tool breaks down, and repairs are made at the time of failure. However, on the other hand, if a critical breakdown occurs, it may cause serious machinery damages. The preventive maintenance aims to prevent the aforementioned problems by scheduling inspection and repair interventions at regular time intervals or operation cycles. In this case, the bigger downside is the waste of time and the replacement costs for components that often are still working. Contrary to previous strategies, PHM relies on the continuous monitoring of equipment health conditions to predict the degradation status, in terms of remaining useful life (RUL), thus supporting more accurate maintenance management decisions.

The prediction of RUL, definable as the “length from the current time to the end of the useful life” as suggested in [5], can be achieved by at least three types of approaches [6]: model-based, data-driven, and hybrid. The model-based approaches (also known as physics-based) refer to mathematical formulations able to model the physical degradation process for the purpose of estimating RUL. In the case of data-driven approaches, instead, RUL is estimated from degradation data collected by monitoring sensors and processed using traditional statistical or machine learning (ML) techniques or even more “advanced” ones based on deep neural networks (DNNs) [7]. However, very often the choice of the appropriate approach depends on the specific problem at hand. Thus, aiming to exploit the strengths of both approaches, data-driven and model-based, they are combined in hybrid approaches by using some kind of fusion scheme [6].

Exploiting the laws of nature to model system degradation, model-based approaches are generally quite accurate. Nevertheless, the implementation of a faithful model for predicting RUL is an expensive and time-consuming process; it may be feasible for simple parts, but may not be for complex components or systems due to the limited understanding of their behavior under all operating conditions. Such disadvantages, combined with the risk of not achieving the desired results, make model-based approaches definitely less attractive than data-driven ones [8].

The various data-driven methods revolve around the processing of features obtained from monitoring sensor data. Consequently, the most important distinction differentiating such methods lies in the way features are obtained, that is, either by traditional handcrafted methods or by learned representations. The methods belonging to the first category utilize representative features, extracted and selected by hand (i.e., handcrafted) on the basis of expert domain knowledge, which are classified or evaluated via regression using appropriate statistical or ML techniques.

The disadvantages of these methods are that handcrafted features are representative only of a specific component or system under certain conditions, while on the other hand, the process of feature extraction and selection is time-consuming and laborious, relying often on strong prior domain knowledge [9]. Moreover, as shown by [10], the performance of ML algorithms is limited by data representation.

In the case of learned representations, on the contrary, representative features of degradation states can be automatically discovered from sensor data by using deep learning (DL) techniques [7,11]. Up to now, a lot of fruitful research results involving many different fields, ranging from image recognition to natural language processing, have been reported in the DL literature [12]. Although, recently, an increasing number of research studies exploiting DL has appeared in the RUL literature, there is still less availability of optimized DL models and architectures compared to other fields.

In the present study, a new DL model based on a convolutional neural network (CNN) is proposed for the RUL prediction of punching tools. The main contributions are (i) representation of punch deformation with depth and normal vector maps (DNVMs) obtained from 3D scan point clouds; (ii) CNN-based RUL prediction with network architecture optimized using genetic algorithm (GA); (iii) validation of the proposed method with real data sets representing three different deformation modes.

## 2. Related Works

Complex real-world data are very useful in many machine-learning applications, including RUL prediction, but they are also cumbersome to process, transmit and store, due to their high-dimensional nature. More effective and low-dimensional features can be obtained from high-dimensional data by using representation learning techniques. The year 2006 marked an important turning point in this research area, since earlier widely used methods such as principal component analysis (PCA) [13,14] and linear discriminant analysis (LDA) [15] gave way to more advanced DL methods [16]. DL architectures, in contrast to shallow ones, are composed of multiple data transformation layers, providing higher hierarchical abstraction levels, and are thus more useful for classification, detection and prediction tasks.

One of the most common DL approach is the stacked sparse auto-encoder (SAE), in which a network of multiple encoder layers is used to transform high-dimensional data into low-dimensional features and, conversely, a network of multiple decoder layers recovers the original data. Specifically, the RUL of an aircraft engine was predicted by Ma et al. [17] by using SAE to extract performance degradation features. Sun et al. [18] addressed the problem of deep transfer learning with SAE networks for predicting the RUL of a cutting tool. They investigated three different transfer strategies, i.e., weight transfer, feature transfer, and weight update, to transfer a trained SAE to a new object tool under operation without providing supervised training information. The authors claimed that deep transfer learning improves performance of RUL prediction also in the case of little historical failure data for training. Ren at al. [19] proposed a DL-based framework for bearing RUL prediction using deep auto-encoder (DAE) and time-frequency-wavelet joint features to represent the bearing degradation process. As the authors pointed out, the advantages offered by the deep autoencoder method were twofold, i.e., automatic feature selection and over fitting problem prevention thanks to reducing network parameters.

Another neural network class arousing considerable research interest in feature learning is represented by the restricted Boltzmann machine (RBM). It is an energy-based neural network with two layers of stochastic binary neurons, one is the visible layer and the other one is the hidden layer. The main issues when dealing with RBMs (even stacked in multiple layers) is the model parameter initialization (e.g., learning rate, momentum, number of hidden units, mini-batch size, etc.) and how to regularize the model to avoid overfitting and improve the learning process. Liao et al. [20] addressed the regularization problem by suggesting a new term allowing an RBM to be trained to output a feature space that better represents degradation patterns in RUL prediction. Although one RBM layer was used, they pointed out that their method can be extended by stacking multiple RBM layers in a deeper neural network architecture.

Haris et al. [21] addressed the problem of finding optimal hyperparameters for a deep belief network (DBN), which is a generative model composed of multiple RMB layers, with the purpose of predicting the RUL of supercapacitors. To this end, they proposed a combination of Bayesian and HyperBand optimization and showed the universality of their model by training it on different degradation profiles with the same hyperparameters.

Aiming to predict the RUL of a complex engineering system whose malfunctions may be caused by multiple faults, Jiao et al. [22] proposed a RUL prediction framework for multiple fault modes consisting of three main modules: DBN-based extraction of degradation features where original data were preprocessed with a gap metric, fault identification under multiple fault conditions via support vector data description (SVDD) monitoring, RUL estimation via the particle filter (PF) and adaptive failure threshold.

Ma et al. [23] assessed the health condition of a bearing rig by using a discriminative DBN model composed of four layers. They obtained the model parameters, i.e., the number of neurons of the two hidden layers and the learning rate, by using the ant colony optimization (ACO) algorithm. The data consisted of vibration signals collected at 10 min intervals with a sampling frequency of 10 kHz. The degradation prediction was formulated as a classification problem with five classes representing the bearing conditions during the evolution process.

Zhang et al. [24] proposed a DBN-based ensemble method for RUL prediction in which multiple DBNs were evolved using a multi-objective evolutionary algorithm integrated with the traditional DBN training technique. They used DBNs with three hidden layers, whose optimization parameters were the number of neurons, the weight cost, and learning rates. They evaluated their method on the turbofan engine degradation problem provided by NASA, i.e., the Commercial Modular Aero-Propulsion System Simulation (C-MAPSS) data sets [25], composed of multivariate temporal data coming from 21 sensors.

Another DL model recently investigated in RUL prediction is the CNN [26], a type of multi-layer feed-forward network originally conceived to recognize visual patterns from images [27], whose feature learning capabilities are enabled through the combination of multiple convolution and pooling layers. Babu et al. [28] reported the first attempt of predicting RUL using CNN-based feature learning combined with linear regression from multi-channel time series data. As the authors pointed out, the multiple layers composing the DL architecture effectively learned local salience representations of multi-channel signals (provided as two-dimensional input) also in different scales. They showed good RUL estimation performance on two publicly available data sets, the NASA C-MAPSS data set and the PHM 2008 Data Challenge data set [29].

Since sensor data involved in RUL estimation are traditionally arranged in a time-series form, either uni- or multi-variate, the use of recurrent neural networks (RNNs) has been suggested to better deal with the sequential nature of these data [30]. However, as it is well-known, RNNs suffer from a vanishing/exploding gradient in the presence of long-term time dependency. To overcome this drawback, Zheng et al. [31] suggested that the RUL be estimated with a long short-term memory (LSTM) network, which is a type of RNN able to effectively model sequential data, without suffering from long-term time dependency problems. To validate their LSTM model for RUL estimation, they used the C-MAPSS data Set, the PHM 2008 Challenge data set, and the Milling data set.

Aiming to exploit the power of CNN to learn discriminative features from two-dimensional inputs, Li et al. [32] proposed a CNN deep learning architecture based on a time window approach able to handle multi-variate temporal information. To validate the effectiveness of their approach, they estimated the RUL of aero-engines using the NASA C-MAPSS data set. The achieved prediction performance was significantly better than the CNN approach presented by Babu et al. [28] and was also comparable with the LSTM approach of Malhotra et al. [33], but employing simpler architecture and lower computing load.

The approaches reviewed so far receive a time series of sensor data as input (e.g., vibration signals, engine data, physical properties, etc.), usually organized as 1D arrays, but often also as 2D arrays in the case of multi-channel data (i.e., multivariate time series). A special case of 2D representation is image data, traditionally used for visual inspection to assess component or system conditions. Recently, image data were used to automatically predict fault or degradation of machine components, with better results for larger data sets [34].

Since large data sets are not always available within PHM applications, the problem of insufficient images for training CNN models has been addressed through transfer learning by Marei et al. [35], using microscope images of a cutting tool flank. Essentially, transfer learning allows knowledge acquired from a similar or different domain to be reused in a new domain. In practice, DL models pretrained on large general-purpose image data sets (e.g., ImageNet [36], ILSVRC: ImageNet Large Scale Visual Recognition Challenge [37], CIFAR-10/CIFAR-100 [38], and so on) can be fine-tuned using available image data to perform predictions on new problems. However, the underlying assumption for transfer learning to work well is that the feature distributions across the two domains are the same.

DL models require an accurate setting of multiple DNN architectural parameters, which is a time-consuming and experience-intensive task. The parameter setting problem has been tackled by Mo et al. [39], adopting an evolutionary algorithm to find the optimal parameter configuration. Furthermore, they proposed a multi-head CNN structure followed by a LSTM network, pointing out the superiority of multi-head CNN models over single-head multi-channel ones, since the former keep the extracted features separate whereas the latter mix them all together losing specialized features. They demonstrated the effectiveness of their solution using time series data from the NASA C-MAPSS data set.

In real-world settings, the collection of machinery health information might be challenging due to some kind of restrictions (e.g., small component size and narrow camera field-of-view, component partially hidden inside the machine, and so on), giving rise to partial or incomplete data. This problem, referred to as the partial observation problem, has been addressed by Li et al. [40] presenting a supervised attention mechanism for feature learning based on CNN and LSTM, followed by a regression layer for estimating the RUL of an industrial cutting wheel from images.

## 3. Materials and Methods

**Figure 1:**
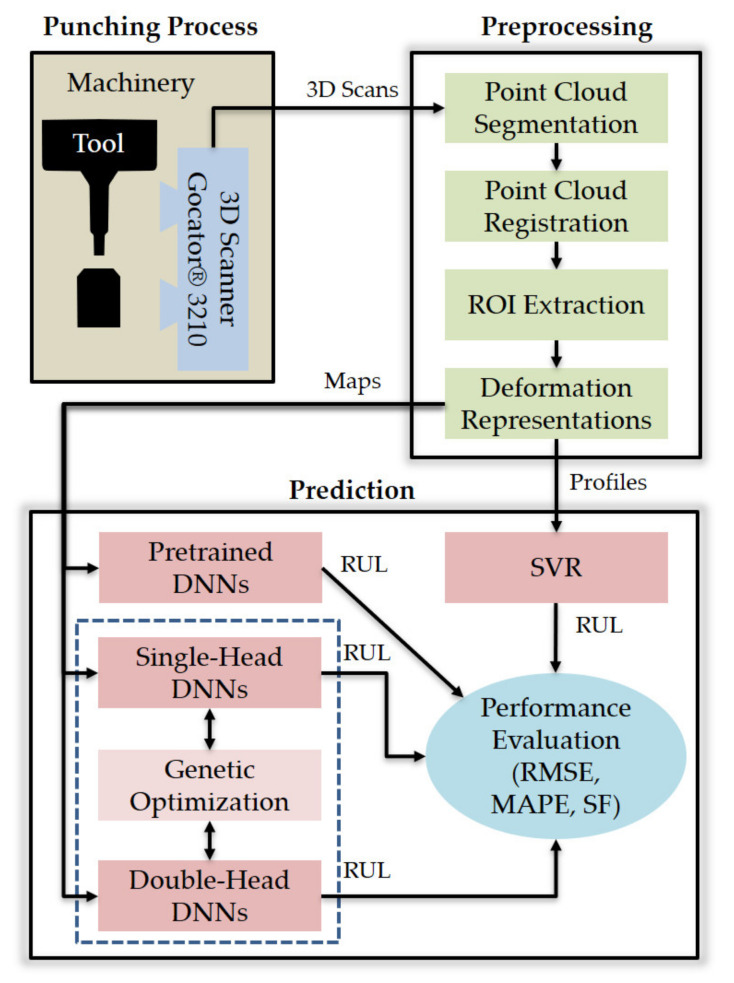
Overview of this research.

This section details the materials and methods used in this study to predict the RUL of a punch tool from 3D scan data. More specifically, the next subsections deal with the following aspects: (1) the data acquisition system and experimental setup, (2) the 3D scanning process (i.e., pre-processing of 3D point clouds and feature extraction), (3) the adopted metrics to evaluate RUL prediction performance, (4) the DNN architectures generated by genetic optimization, (5) the genetic optimization technique, and (6) the support vector regression (SVR) approach used as classic ML benchmark. The overview of this research is provided in Figure 1, the components of which are detailed in the following subsections.

### 3.1. Experimental Setup and Data Acquisitions

**Figure 2:**
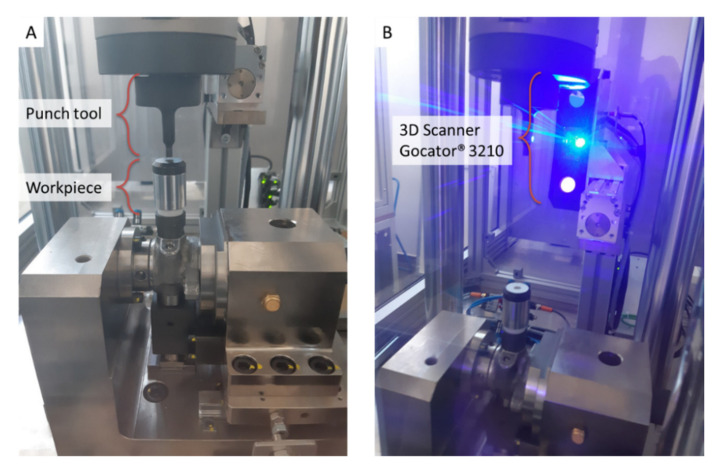
Punching machinery: (**A**) punch tool and pump workpiece, (**B**) Gocator^®^ 3210 3D scanning sensor.

The present study focuses on RUL prediction of punch tools mounted on a punching machine, such as the one shown in Figure 2, used to process pump workpieces by making a punch on their upper end. The RUL prediction is based on 3D scan data acquired by a Gocator^®^ 3210 (LMI Technologies Inc., Burnaby, BC, Canada) [41] sensor, tightly clamped on the punching machine structure (Figure 2B). The 3D scan sensor, equipped with a stereo camera of two megapixels and a blue LED projector, provides 3D point clouds in a single snapshot for accurate noncontact measurements down to 35 μm.

**Figure 3:**
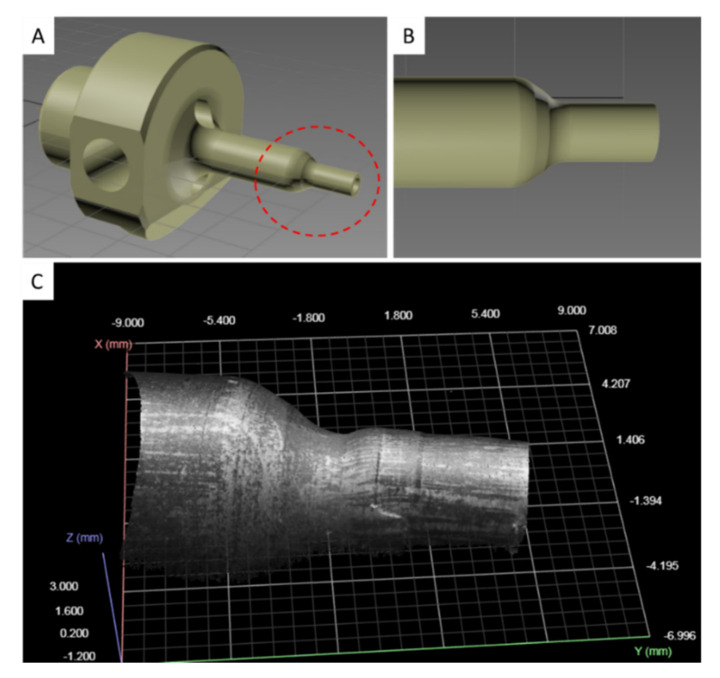
Punch tool 3D model. (**A**) Full view of the punch tool with working region circled with a dashed red line. (**B**) Detail of the working region. (**C**) 3D point cloud with grayscale patches of the working region obtained from a 3D scan snapshot.

The punch tool consists of two cylindrical ends, of which the one having a larger diameter serves to clamp it to the machine, while the other is the working region. The 3D model of the punch tool is provided in Figure 3, in which the working region is highlighted by a dashed red line (Figure 3A). During the machining cycles, the working surface of the punch (Figure 3B) undergoes progressive deformations (Figure 3C). Three-dimensional scans of these surface deformations, suitably processed using ML algorithms, can be exploited to predict the RUL of the punch tool.

During the experimentation phase, three identical punch tools were brought to the end of their life cycle, subjecting them to different loads. The first punch tool, P1, was operated with an incremental load ranging from a minimum of 10 kN to a maximum of 15 kN, obtaining a total of 714 scans performed every 50 pressing cycles. The second punch tool, P2, was tested with an incremental load ranging from 20 to 28 kN, for a total of 521 scans performed every 60 pressing cycles. The third punch, P3, was subjected to an incremental load ranging from 15 to 20 kN, generating a total of 779 scans captured every 50 pressing cycles. In such a way, a total number of 2014 scans was produced in approximately six months.

### 3.2. D Scan Preprocessing

**Figure 4:**
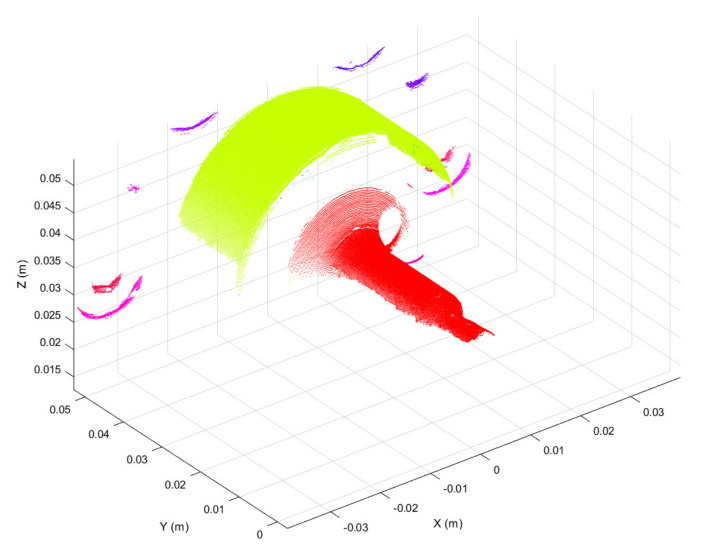
3D point cloud segmented using the Euclidean distance between 3D points from different clusters. Segmented clusters are represented in different colors.

**Figure 5:**
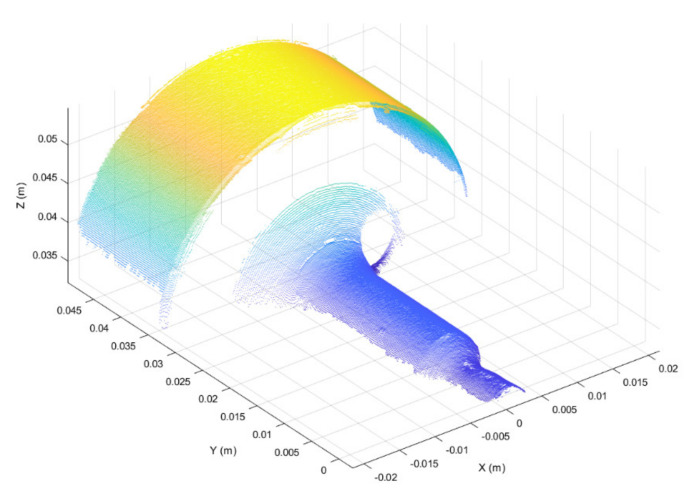
Filtered 3D point cloud after segmentation. Two main segments are visible.

Three-dimensional scans obtained as discussed above were preprocessed in the form of 3D point clouds. Due to reflections from the metallic surface, raw 3D point clouds may be corrupted by artefacts, i.e., spurious 3D points. To overcome this issue, the first preprocessing step was to segment each raw point cloud into clusters, considering a minimum Euclidean distance between 3D points from different clusters, as represented in Figure 4. Then, the clusters were filtered based on the number of 3D points, keeping the two clusters with the greater number of points. The resulting 3D point cloud, provided in Figure 5, is composed of two main segments. The points located at y < 0.01 form the working surface, whereas those located at y > 0.03 represent the so-called best-fit surface used in the following step for point cloud registration. Although the 3D scan system is firmly clamped to the machine structure, continuous vibrations can produce slight misalignments between 3D point clouds. A rigid registration step was utilized to correct such misalignments.

The semi-cylindrical surface with the largest diameter, shown in Figure 5 (i.e., 3D points with y > 0.03), was used as the best-fit reference surface for registration, since this surface was the least subject to deformation during punching operations. The iterative-closest-point (ICP) algorithm, originally suggested by Besl and McKay [42], was used to register the best-fit surfaces of segmented point clouds.

Substantially, the ICP algorithm is an optimization process whose main goal is to find the best local (in a least-square sense) rigid transformation by means of singular value decomposition (SVD) [43]. More specifically, the iterative process consists of the following main steps: (1) projection of the two point clouds under registration, (2) estimation of the optimal rigid transformation via SVD, (3) application of the transformation to a subset of randomly selected points, (4) evaluation of the alignment via least median estimator [44], (5) if the alignment error is smaller than a prefixed threshold, the rigid transformation is applied to all the point clouds, otherwise, the above steps are repeated. The pseudocode of the registration procedure is reported in Algorithm 1.
**Algorithm 1.** Point cloud registration1.$P_{0}\leftarrow \left\{P_{i}^{R}\in {\mathbb{R}}^{3},i=1,\ldots ,N_{0}\right\}$;//Reference point cloud2.$P\leftarrow \left\{P_{i}\in {\mathbb{R}}^{3},i=1,\ldots ,N\right\}$;//Data point cloud to be registered ($N>N_{0}$)3.ε=0.01;//Tolerance between consecutive iterations4.MaxIterations=100;//Maximum number of iterations 5.for $i=1;i\leq N_{0};i++$
6.   ${\hat{P}}_{i}^{0}\leftarrow P_{i}^{0}-\frac{1}{N_{0}}\overset{N_{0}}{\underset{j=1}{\sum }}P_{j}^{0}$; //Normalization of $P_{0}$7.end for8.for k=1; k≤MaxIterations; k++
9.   $P\leftarrow Projectiection\;of\;P\;onto\;P_{0}$;//Matching subset (from now $\left|P\right|=N_{0}$)10.   for $i=1;i\leq N_{0};i++$
11.      ${\hat{P}}_{i}\leftarrow P_{i}-\frac{1}{N_{0}}\overset{N_{0}}{\underset{j=1}{\sum }}P_{j}$; //Normalization of P12.    end for13.   *Calculate the SVD decomposition of the matrix* $H=\overset{N_{0}}{\underset{j=1}{\sum }}{\hat{P}}_{j}^{0}{\left({\hat{P}}_{j}\right)}^{T}=U\Lambda V^{T}$;14.   $R\leftarrow VU^{T}$;//Rotation matrix15.   $T\leftarrow \frac{1}{N}\overset{N_{0}}{\underset{j=1}{\sum }}{\hat{P}}_{j}-R\frac{1}{N}\overset{N_{0}}{\underset{j=1}{\sum }}{\hat{P}}_{j}^{0}$;//Translation matrix16.    if $\overset{N_{0}}{\underset{j=1}{\sum }}{\hat{P}}_{j}^{0}-\left(R{\hat{P}}_{j}+T\right)\leq \varepsilon$ //Termination condition17.       break;18.    end if19.end for

**Figure 6:**
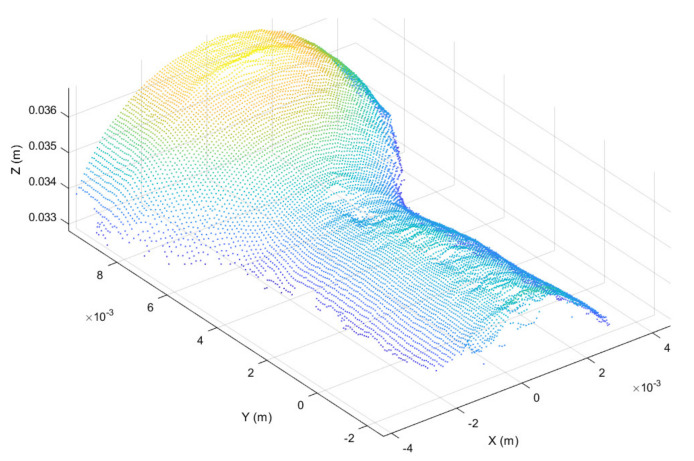
Working surface cropped from registered point cloud.

**Figure 7:**
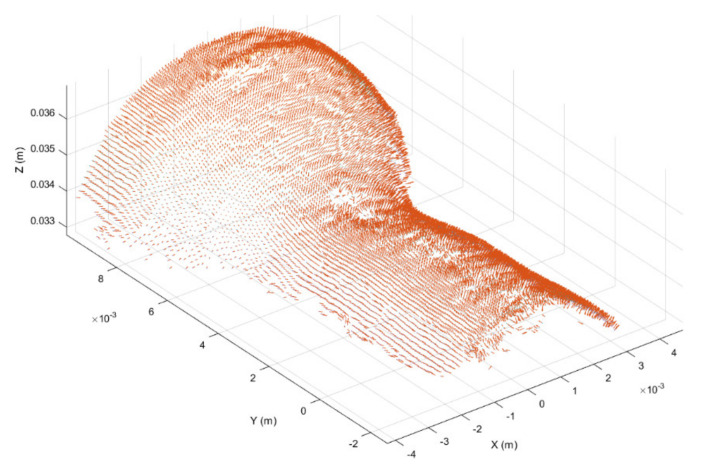
Surface normal vector representation of the working region obtained from the cropped point cloud provided in Figure 6.

Once registered, the point cloud was cropped to take only the working region, thus highlighting the surface deformation, as shown in Figure 6. In addition, in order to further highlight the working surface deformations, for each point belonging to the cropped point cloud the surface normal vector was considered [45], obtaining the normal representation shown in Figure 7.

**Figure 8:**
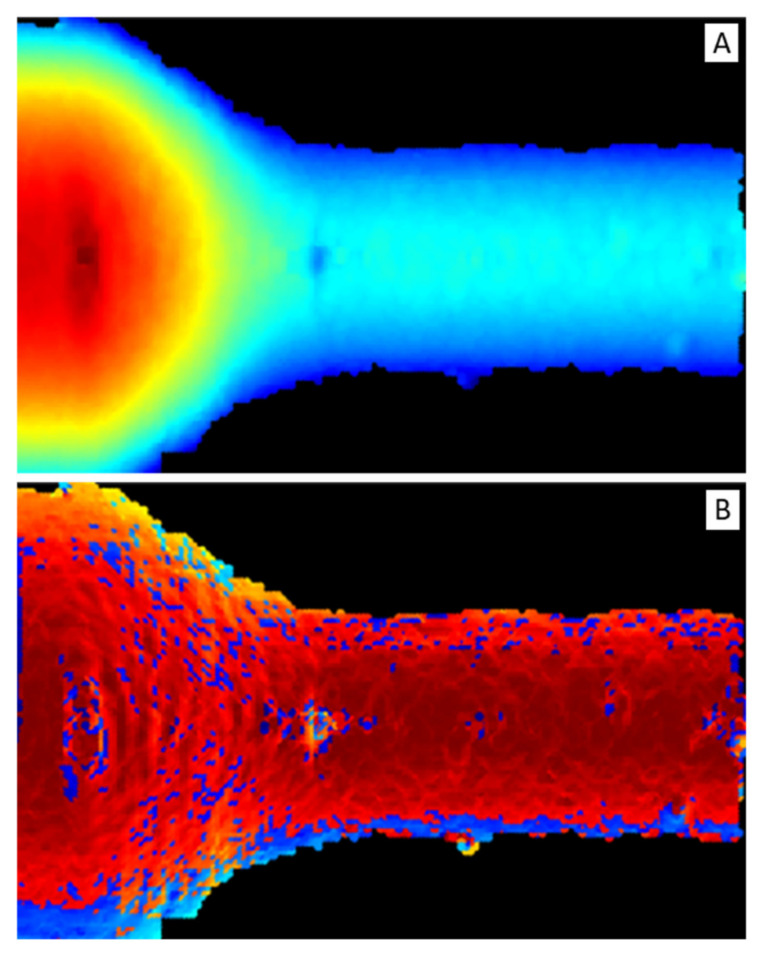
(**A**) Depth map. It is a false-color image: red color represents short distances and blue long ones. (**B**) Normal map. It is a false-color image: dark-red color represents vectors parallel to *z*-axis (i.e., pointing out of the image plane) and light-blue perpendicular ones.

To exploit most of the DNN feature extraction capabilities, depth and normal vector representations were transformed into two-dimensional maps, reported in Figure 8, i.e., depth map (Figure 8A) and normal vector map or normal map (Figure 8B), respectively.

**Figure 9:**
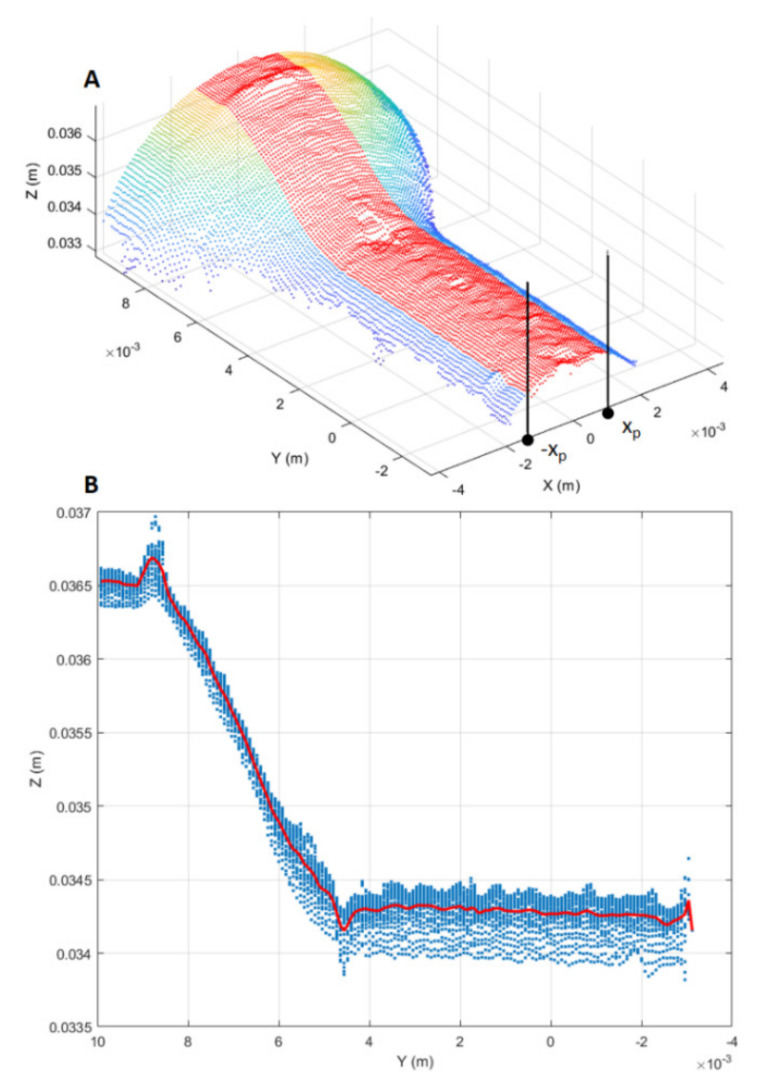
Longitudinal profile extraction. (**A**) Longitudinal point-cloud region (red points) from which the longitudinal profile is estimated. (**B**) Estimated longitudinal profile (red curve) from the point cloud projection on the YZ plane.

The RUL prediction based on the DNN was compared with that based on traditional ML methods (e.g., SVR). To do so, alongside the two-dimensional deformation representations mentioned above, one-dimensional representations were also considered, i.e., longitudinal profiles of the punch tool. The extraction process of longitudinal profiles, shown in Figure 9, consisted of three main steps. Firstly, a profile region was selected from the cropped point cloud by taking points $P=\left(x,y,z\right)$ such that $x\in \left[-x_{p},x_{p}\right]$ (Figure 9A).

Secondly, the selected point-cloud region was projected onto the YZ plane. Thirdly, the longitudinal profile was estimated averaging the projected region along the *Z*-axis direction (Figure 9B).

### 3.3. Evaluation Metrics

Three different evaluation metrics were adopted in this study, i.e., scoring function (SF), root mean square error (RMSE), and mean absolute percentage error (MAPE). The first two metrics were selected since they are commonly adopted in the literature on RUL prediction [32], while the third was considered as it allows for more subtle evaluations than the other two.

Given a total number N of sampled machining cycles (i.e., 3D scans), let $p_{i}$ be the RUL at the machining cycle i, $p_{i}^{\prime }$ the estimated version of $p_{i}$, and $E_{i}=p_{i}^{\prime }-p_{i}$ the prediction error, the SF is defined as follows:

(1) $$SF=\sum_{i=1}^{N}f_{i},\;\mathrm{where}\;f_{i}=\left\{\begin{array}{c} e^{-\frac{E_{i}}{13}}-1,\;\;iif\;E_{i}<0\\ e^{\frac{E_{i}}{10}}-1,\;\;iif\;E_{i}\geq 0 \end{array}\;\;\right..$$

Furthermore, the RMSE is defined as follows:

(2) $$RMSE=\sqrt{\frac{1}{N}\sum_{i=1}^{N}E_{i}^{2}},$$

Finally, MAPE is given by:

(3) $$MAPE=\frac{1}{N}\sum_{i}^{N}\left|\frac{E_{i}}{p_{i}}\right|,\;\mathrm{for}\;p_{i}>0.$$

### 3.4. DNN Architectures

**Figure 10:**
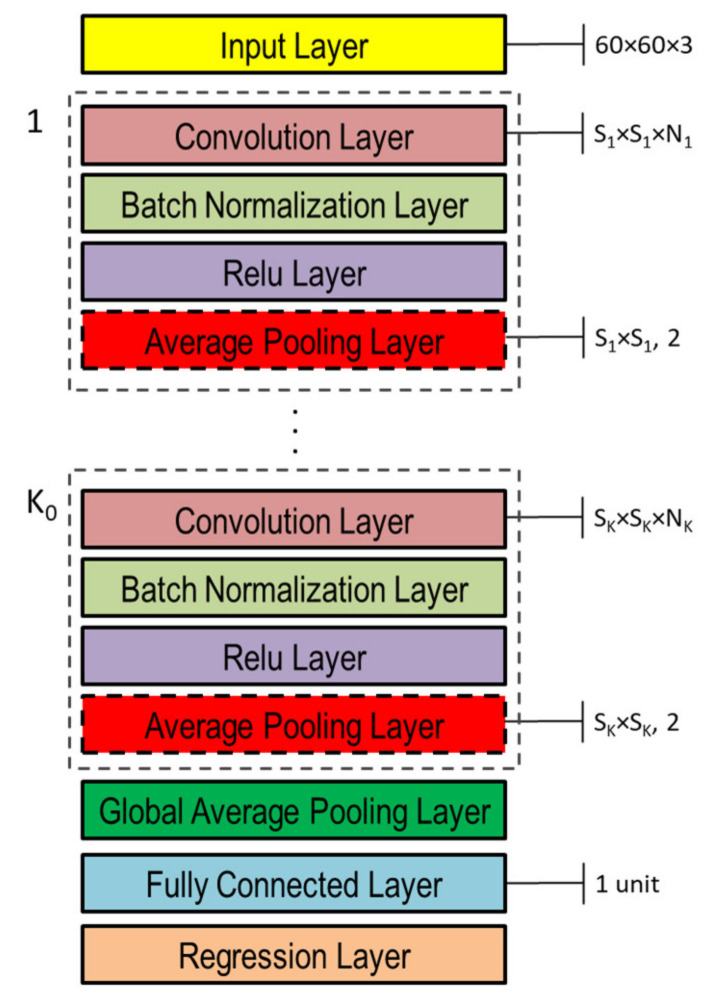
Single-head general architecture of CNN-based DNN for processing either depth or normal maps provided as color image inputs.

**Figure 11:**
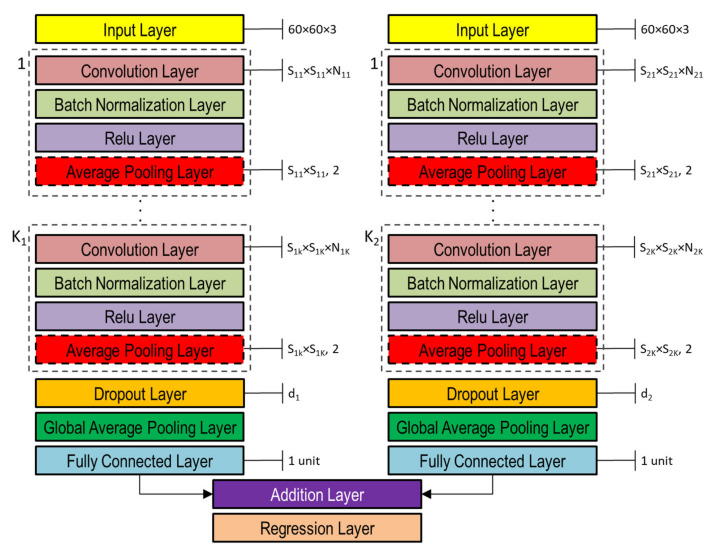
Double-head general architecture of CNN-based DNN for processing both depth and normal maps provided as color image inputs.

The DNNs used in this study to capture the representation information from preprocessed inputs, i.e., depth and normal maps provided as false-color images (Figure 8), were based on CNN [46]. Both single- and double-head DNNs were investigated, whose general architectures are shown in Figure 10 and Figure 11, respectively. Basically, they are composed of an input layer receiving depth or normal maps, resized to 60 × 60 pixels color (3-channels) images, followed by $K_{0}$ ($K_{1}$ and $K_{2}$ in the double-head case) blocks including the following four layers: (1) convolution, (2) batch normalization, (3) rectified linear unit (ReLU), and (4) global average pooling.

CNN feature learning is based on the convolution operation implemented by applying a kernel to input images (i.e., local receptive field) whose response provides the so-called feature map. Let $I=\left(I_{1},I_{2},I_{3}\right)$ be an input image, W a kernel of size (a square kernel was considered in this study, but in general it may have a rectangular size) whose $s^{2}$ weights are adjusted in a feedforward way, the feature map computed at $\left(x,y\right)\in I$ is given as follows:

(4) $$F\left(x,y\right)=\sum_{i,j=1}^{s}I_{h}\left(x-i,y-j\right)W\left(i,j\right),\;\mathrm{with}\;h=1,2,3;$$

where the summation is the convolution operation as the kernel Q slides over the image channel $I_{h}$.

During the feedforward process, the output of a generic convolution layer at $\left(x,y\right)$ is given as follows:

(5) $$O_{h}\left(x,y\right)=\varphi \left(\sum_{i,j=1}^{s}I_{h}\left(x-i,y-j\right)W\left(i,j\right)+b\right),\;\mathrm{with}\;h=1,2,3;$$

where $\varphi \left(\cdot \right)$ is the activation function used to introduce nonlinearity to feature maps, and b is the bias term. In this study, the ReLU activation function was used which performs an element-wise threshold operation, i.e., sets to zero any input value less than zero:

(6) $$\varphi \left(\nu \right)=\left\{\begin{array}{c} \nu ,\;\;\;iif\;\nu \geq 0\\ 0,\;\;\;iif\;\nu <0 \end{array}\right.\;.$$

The batch normalization layer, inserted between convolution and nonlinear activation (ReLU) layers, allows training to be sped up and the network to be regularized, reducing initialization sensitivity, i.e., it facilitates the convergence to good local minima without cumbersome initial parameter setting. Given an input element $z_{ij}$ the batch normalization layer provides the following normalization:

(7) $${\hat{z}}_{ij}=\frac{z_{ij}-\mu }{\sqrt{\sigma^{2}+\epsilon }},$$

where μ and σ are, respectively, mean and variance estimated over spatial, time and observation dimensions for each channel independently, whereas ϵ is a constant used to improve numerical stability if variance is too small.

The average pooling layer performs down-sampling by providing average values of its input as output and thus reducing the connection number to the next layer, which helps to mitigate overfitting. The pool size adopted in this study was the same as the corresponding convolution layer in each CNN block, whereas the stride (i.e., step size with which the pooling layer scans through the input) was fixed at 2. The effect of the dropout layer is to turn off (set to zero), with probability p, a certain number of input elements randomly chosen. Such dropout operation has been shown to help prevent network overfitting [47]. In this study, the dropout layer was adopted only in the double-head case with $p=d_{1}$ and $p=d_{2}$ in the two heads, respectively (Figure 11).

The global average pooling layer provides further down-sampling by fully averaging the feature map. It is usually used before the final fully connected layer to reduce the size of activations, i.e., less weights, thus leading to a lower network size. The purpose of fully connected layers is to combine features to identify larger patterns. Thus, all the neurons in a fully connected layer are connected to all the neurons of the previous layer.

In case of classification problems, the last fully connected layer provides the features to perform classification, thus, its output size is equal to the number of classes being classified. In case of regression problems, the output size is equal to the number of regression variables, which is one in this study. In both single- and double-head architectures, the continuous RUL value was finally estimated in the regression layer by minimizing the loss (i.e., not-normalized half mean squared error), which in the case of this study with only one regression variable, reduces to $E_{i}^{2}$.

Generally, the learning process is cast as an optimization problem of minimizing the loss function. In this study, the stochastic gradient descent (SGD) [48] was used as an optimization scheme. In SGD training, a mini-batch is stochastically selected at each time step, instead of using the entire training set, thus improving computing speed. Two relevant SGD parameters are initial learning rate η and momentum λ. If η is too low, the training process takes a long time, whereas if it is too high, the training might result in being suboptimal or divergent.

The momentum is a technique used in conjunction with SGD that adjusts the contribution of gradients at previous steps to determine the direction to proceed, instead of using only the gradient at the current step. If λ is equal to zero there is no contribution from previous steps, whereas if λ is one the contribution from previous steps is maximal. In this study, the network hyperparameters $\left(S_{1},N_{1},\ldots ,S_{K},N_{K},\eta ,\lambda \right)$ in the single-head case and $\left(S_{11},N_{11},\ldots ,S_{1K},N_{1K},d_{1},S_{21},N_{21},\ldots ,S_{2K},N_{2K},d_{2},\eta ,\lambda \right)$ were determined using a genetic optimization algorithm as discussed in the following subsection. Note that the average polling layer included in each CNN block is optional and its inclusion or exclusion was also considered among the network hyperparameters subject to optimization through binary array variables, as detailed in the following subsection.

In addition to the architectures shown in Figure 10 and Figure 11, pretrained networks were also evaluated. The adoption of pretrained models offers multiple advantages, such as the possibility to exploit complex models without having to train them from scratch, even when little training data are available (used to fine-tune the pretrained model) without running into overfitting problems, commonly found in the presence of small training data sets. The technique underlying pretrained models is called transfer learning [49] and, basically, allows the knowledge already learned from one domain to be applied to another.

However, the transfer of knowledge from one domain to another is not always feasible. When the source domain is not sufficiently related to the destination domain, or when the transfer methodology is not able to take advantage of relationships between domains, this can lead to negative transfer [49]. For that reason, in this study, the most popular state-of-the-art pretrained models reported in Table 1 were evaluated, and their prediction performance was compared with that of network architectures discussed above. Since such pretrained networks are designed for classification problems, they were adapted by substituting the last three layers, i.e., the global average pooling layer, the softmax layer, and the classification layer, with a fully connected layer with one output neuron followed by a regression layer.

### 3.5. Genetic Optimization

The parameters of the network architectures shown in Figure 10 and Figure 11 were optimized by means of a genetic optimization technique. GAs are population-based optimization methodologies that take their cue from the evolutionary process of living beings, i.e., the metaphor of natural biological evolution [64]. GAs iteratively implement a series of operations to manipulate populations of candidate solutions (i.e., chromosomes) to produce new solutions by means of genetic functionals such as reproduction, crossover, and mutation. Ultimately, they are inspired by Darwin’s theory of evolution [65] and relative principles of reproduction, genetic recombination, and survival of the fittest. The population of chromosomes (i.e., candidate solutions) is evaluated through the attribution of a score carried out through a so-called fitness function, the formulation of which depends on the specific optimization problem.

In this study, the fitness function f built network architectures and evaluated them, providing as output the MAPE value obtained from testing. Thus, the optimization problem was formulated as follows:

(8) $$\mathrm{minimize}f\left(z\right),\;\mathrm{with}\;z=\left(z_{1},z_{2},\ldots ,z_{M}\right)\;\mathrm{such}\;\mathrm{that}\;z_{i}^{L}\leq z_{i}\leq z_{i}^{U},\;i=1,\ldots ,M,$$

where $\left(z_{1},z_{2},\ldots ,z_{M}\right)$ are optimization variables, continuous or integer valued, bounded between $\left(z_{1}^{L},z_{2}^{L},\ldots ,z_{M}^{L}\right)$ and $\left(z_{1}^{U},z_{2}^{U},\ldots ,z_{M}^{U}\right)$, respectively, defining candidate network architectures as better explained in the following. The number of optimization variables was M=5 for single-head architectures (Figure 10) and M=10 for double-head architectures (Figure 11). For both architectures, the number of CNN blocks ranged from four to six, i.e., $K_{i}\in \left\{4,5,6\right\},\;i=1,2,3$.

In the single-head case, the optimization variables were $z=\left(x_{1},x_{2},x_{3},x_{4},x_{5}\right)$, where $x_{1}\in \mathbb{N}$ represented the filter sizes, $x_{2}\in \mathbb{N}$ specified the number of filters, $x_{3}\in \mathbb{N}$ the presence or not of average pooling layers, $x_{4}\in \mathbb{R}$ the initial learning rate, and $x_{5}\in \mathbb{R}$ the momentum. In the double-head case, instead, the optimization variables were $z=\left(y_{1},y_{2},y_{3},y_{4},y_{5},y_{6},y_{7},y_{8},y_{9},y_{10}\right)$, and $y_{1},y_{2},y_{3}\in \mathbb{N}$ represented the filter sizes, number of filters, and presence or not of average pooling layers for the first head, whereas $y_{4},y_{5},y_{6}\in \mathbb{N}$ were the filter sizes, number of filters, and presence or not of average pooling layers for the second head. $y_{7}\in \mathbb{R}$ was the initial learning rate, $y_{8}\in \mathbb{R}$ the momentum, $y_{9}\in \mathbb{R}$ the dropout probability for the first head, and $y_{10}\in \mathbb{R}$ the dropout probability for the second head.

Regarding the convolutional filter sizes, i.e., $x_{1}$ (single-head) or $y_{1}$ and $y_{4}$ (double-head) variables, odd square dimensions ranging from 3 × 3 to 29 × 29 were evaluated by considering all possible combinations taking $K_{i}$ (i=0,1,2) at a time. For example, in the case of $K_{0}=4$, $x_{1}=1$ corresponded to $\left(S_{1},S_{2},S_{3},S_{4}\right)=\left(3,5,7,9\right)$, $x_{1}=2$ to $\left(S_{1},S_{2},S_{3},S_{4}\right)=\left(3,5,7,11\right)$, $x_{1}=3\;$ to $\left(S_{1},S_{2},S_{3},S_{4}\right)=\left(3,5,7,13\right)$, etc., and $x_{1001}$ to (23,25,27,29); after that, it continued in reverse order, i.e., $x_{1002}$ corresponded to (29,27,25,23), $x_{1003}$ corresponded to (29,27,25,21), and so on. Regarding the number of filters, i.e., $x_{2}$ (single-head) or $y_{2}$ and $y_{5}$ (double-head) variables, power of two between 8 and 256 were considered in incremental order. Thus, for example, in the case $K_{0}=5,$ $x_{2}=1$ gave $\left(N_{1},N_{2},N_{3},N_{4},N_{5}\right)=\left(8,8,8,8,8\right)$, $x_{2}=2$ gave $\left(N_{1},N_{2},N_{3},N_{4},N_{5}\right)=\left(8,8,8,8,16\right)$,$x_{2}=3$ gave $\left(N_{1},N_{2},N_{3},N_{4},N_{5}\right)=\left(8,8,8,8,32\right)$, and so on.

Since the average pooling layers, depicted in Figure 10 and Figure 11 with dashed lines, were optional, their presence or absence were regulated by variables $x_{3}$ (single-head) or $y_{3}$ and $y_{6}$ (double-head), representing the configurations of a binary array ${\left\{0,1\right\}}^{M_{i}}\;$, where 1 in j-th position (with $j=1,\ldots ,M_{i}$) indicated the presence of average pooling layer at the end of the j-th CNN block. For example, in the case of $K_{0}=6$, $x_{3}=1$ corresponded to $\left(0,0,0,0,0,0\right)$, $x_{3}=2$ corresponded to $\left(0,0,0,0,0,1\right)$, $x_{3}=3$ to $\left(0,0,0,0,1,1\right)$, and so on. All previously discussed optimization variables are summarized in Table 2.

In this study, both suggested DNNs (Figure 10 and Figure 11) and pretrained ones (Table 1) were implemented and evaluated using the MathWorks^®^ Deep Learning Toolbox (v 14.2, R2021a, MathWorks Inc., Natick, MA, USA) [66]; whereas, genetic optimization was performed using the MathWorks^®^ Optimization Toolbox (v 9.1, R2021a, MathWorks Inc., Natick, MA, USA) [67].

### 3.6. SVR Based Estimation

As a further comparison, the previously presented DNN-based models were compared with more traditional ML methods such as SVR. Since the presence of irrelevant or redundant information could slow down or make prediction algorithms less accurate, it is necessary, before the learning model, to distinguish between relevant and unnecessary features. For this reason, the first step was to reduce the dimensionality of the profile data (Figure 9B) using the PCA approach [68].

The profile data were represented in YZ plane by curves $\Psi_{k}=\left\{\left(y_{i}^{k},z_{i}^{k}\right)\in {\mathbb{R}}^{2},\;i=1,\ldots ,N_{P_{k}}\right\}$, k=1,…,N, with $N_{P_{k}}$ typically ranging between 156 to 164 depending on the specific point cloud considered. After the PCA application, the reduced profile data were given by ${\bar{\Psi }}_{k}=\left\{\left({\bar{y}}_{i}^{k},{\bar{z}}_{i}^{k}\right)\in {\mathbb{R}}^{2},\;i=1,2\right\}$, since the percentage of variance explained by the first two principal components was 100%. Ultimately, profile feature data used to train and test the SVR model was written as follows:

(9) $$P=\left[\begin{array}{c} \begin{array}{cc} \begin{array}{cc} {\bar{y}}_{1}^{1} & {\bar{z}}_{1}^{1} \end{array} & \begin{array}{cc} {\bar{y}}_{2}^{1} & {\bar{z}}_{2}^{1} \end{array} \end{array}\\ \vdots \\ \begin{array}{cc} \begin{array}{cc} {\bar{y}}_{1}^{N} & {\bar{z}}_{1}^{N} \end{array} & \begin{array}{cc} {\bar{y}}_{2}^{N} & {\bar{z}}_{2}^{N} \end{array} \end{array} \end{array}\right]\in {\mathbb{R}}^{N,4}.$$

To achieve a good compromise between processing speed and accuracy, in this study, the epsilon-insensitive SVR (i.e., ε− SVR) [69,70] was adopted, in which the epsilon parameter controls the amount of error allowed in the model. Given training data $P_{i}={\left({\bar{y}}_{1}^{i},{\bar{z}}_{1}^{i},{\bar{y}}_{2}^{i},{\bar{z}}_{2}^{i}\right)}^{T}\in {\mathbb{R}}^{4}$, the goal is to find a function $g\left(P_{i}\right)$ that deviates from RUL values $p_{i}\in \mathbb{R}$ by an amount no greater than ϵ while at the same time being as flat as possible.

In the linear case, assuming that the training data set is composed of $N_{T}<N$ profiles $P_{i}$ and corresponding RUL values $p_{i}$ with $i=1,\ldots ,N_{T}$, the linear function takes the form $g\left(P_{i}\right)=\langle \pi \cdot P_{i}\rangle +b$, where 〈·〉 is the dot product, and $\pi \in {\mathbb{R}}^{4}$ such that 〈π·π〉 is minimum to ensure flatness. The problem can be stated in term of convex optimization as follows:

(10) $$\mathrm{minimize}\;{∥\pi ∥}^{2},\;\mathrm{subject}\;\mathrm{to}\;\forall i=1,\ldots ,N_{T}\;:\;\left|p_{i}-\left(\pi \cdot P_{i}+b\right)\right|\leq \epsilon .\;$$

Since a function f satisfying these constraints for all points may not exist, in practice, slake variables $\left(\xi_{i},\xi_{i}^{*}\right)$ are introduced, analogously to the concept of “soft margin” in SVM. With the addition of the slake variables problem (9) becomes [66]:

(11) $$\mathrm{minimize}\;\pi^{2}+C\overset{N_{T}}{\underset{i=1}{\sum }}\left(\xi_{i}+\xi_{i}^{*}\right),\;\mathrm{subject}\;\mathrm{to}\;\forall i=1,\ldots ,N_{T}\;:\;\left\{\begin{array}{c} p_{i}-\pi \cdot P_{i}-b\leq \epsilon +\xi_{i}\\ \pi \cdot P_{i}+b-p_{i}\leq \epsilon +\xi_{i}^{*}\\ \begin{array}{c} \xi_{i}\geq 0\\ \xi_{i}^{*}\geq 0 \end{array} \end{array}\right.,\;$$

where the positive parameter C controls the penalty imposed on observations that fall outside the ϵ margin, playing a regularizing role to prevent overfiting.

In the nonlinear case, the dot product is replaced with a kernel function $G\left(\cdot ,\cdot \right)$ which maps training data to a high-dimensional space. Some popular kernel functions evaluated in this study are reported in Table 3.

## 4. Results

The performance results of the genetically optimized network architectures are provided in Table 4. In the single-head case, the convention used for the model name is a prefix “go” which stands for genetically optimized, followed by the number of CNN blocks and the suffix “normal” or “depth” depending on the type of map the model was tested on. Thus, for instance, the name of the model genetically optimized with four CNN blocks and tested on normal maps is “go4normal”. Instead, in the double-head architectures, since they were tested on both normal and depth maps, the naming convention consists of the suffix “go” followed by the number of blocks for the two heads, for example, “go4+4” indicates a model with four blocks for each head. In addition to the three metrics defined in (1), (2), and (3), the last column of Table 4 provides the time required to train each model.

The population size was 50 in single-head architectures (5 optimization variables) and 200 in double-head ones (10 optimization variables), for a total number of 10,000 and 40,000 iterations, respectively. For each candidate architecture, the model was trained for 100 epochs, randomizing the validation data set to each epoch. To achieve the final performance, the results of the top 100 architectures based on the fitness function (i.e., the MEPA metric) were averaged.

The pretrained models (Table 1) tested on normal and depth maps are provided in Table 5 and Table 6, respectively. The last columns of these tables report the fine-tuning time (FTT), i.e., the time elapsed to fine-tune each pretrained model for 30 epochs. Also in this case, performance results were averaged by repeating training and testing 100 times for each model.

As regards the classical approaches based on SVR, three kernels, i.e., linear, Gaussian and polynomial of order 3, 4, 5, and 6, were tested. The performance results obtained with the approaches based on SVR are reported in Table 7. In this study, SVR was considered as a benchmark for evaluating the goodness of DNN-based models.

**Figure 12:**
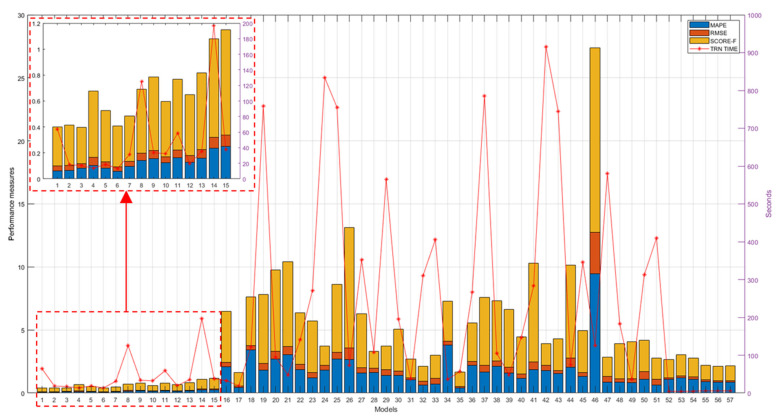
Performance measures (**left** *y*-axis) and TTs/FTTs (**right** *y*-axis) of all evaluated models.

A comprehensive overview of all achieved results is shown in Figure 12. As can be seen from this figure, the models that performed best are the genetically optimized ones (numbers from no. 1 to 15), while most of the pretrained models (from no. 16 to 51) performed worse than the SVR algorithms (from no. 52 to 57), with the exception of the pretrained models, googlenet (no. 17), vgg16 (no. 32), vgg19 (no. 33), on maps of normal vectors and the pretrained models, googlenet (no. 35), alexnet (no. 49), and vgg19 (no. 51), on depth maps.

Figure 12 also reports training times (TTs) of genetically optimized and SVR-based models and FTTs of pretrained models, revealing on average longer times for the pretrained models (from no. 18 to 51), on average shorter times for the genetically optimized models (from no. 1 to 15), and very short times for the SVR models (from no. 52 to 57). Note that, to facilitate reading, the numbering reported on the *x*-axis in Figure 12 matches the model numbers reported in the first column of the tables from Table 1, Table 2, Table 3, Table 4, Table 5, Table 6, Table 7, Table 8 and Table 9, for a total of 57 examined models.

Finally, the network configurations of genetically optimized single- and double-head DNN architectures are reported in Table 8 and Table 9, respectively. The reported parameters refer to best-fit models resulting from the genetic optimization process. The last columns show the number of learned parameters (learnables) of each architecture. The genetic optimization process lasted an average of 53 h for each single-head architecture and approximately 632 h for each dual-head architecture. The total duration of the genetic optimization process was approximately 250 days on a computer system equipped with a graphics processing unit (GPU) and configured as follows: Intel^®^ Core™ i7-5820K CPU @ 3.30 GHz, 16 GB DRAM, and NVIDIA GeForce GTX TITAN X GPU (Maxwell family) with 12 GB GRAM.

## 5. Discussion

The use of profilometric scanning sensors allows surface deformations to be appreciated with micrometric precision in the form of 3D point clouds. On the other hand, the organization of 3D point clouds into bidimensional image-like maps, as proposed in this study, enables us to make the most of the potential of CNN-based DNN architectures, originally designed to process image data. Furthermore, in the case of RUL depending on surface deformations, two-dimensional maps offer the advantage of representing the state of system (i.e., punch tool in this study) deterioration in a cumulative way. Therefore, under such conditions, the RUL can be reliably estimated from a single image, that is from a single depth or normal map.

Pretrained networks with transfer learning are advantageous since they allow small training data sets to be dealt with and the often-cumbersome process of generating problem-specific networks to be overcome. However, they are not always suitable, especially when data distributions are very dissimilar between source and target domains.

The definition of the most suitable DNN architecture for the problem under consideration, however, is not an easy task. In general, it involves identifying various configuration parameters (hyperparameters) through a trial-and-error process. The transfer learning method offers the indisputable advantage of simplifying this often cumbersome process of generating ad-hoc DNN architectures. In addition, pretrained models require the use of a small amount of training data for fine-tuning, allowing the additional problem of reduced amount of training data to be addressed [35].

Keeping in mind the aforementioned advantages, in this study, the transfer learning technique was evaluated in correspondence with the main pretrained models, as reported in Table 1, with both depth and normal maps. However, the performance results obtained were generally lower than the more traditional SVR approach taken as a reference (Figure 12). Only the pretrained models googlenet (MAPE equal to 0.452 with normal maps and 0.416 with depth maps), vgg16 (MAPE = 0.615 with normal maps), vgg19 (MAPE equal to 0.698 with normal maps and 0.648 with depth maps), and alexnet (MAPE = 0.822 with depth maps) performed slightly better than the SVR approach (MAPE = 0.857 with polynomial kernel of order 6), but requiring on average 35 times more time for fine-tuning than SVR requires for training.

In this study, the problem of defining ad-hoc (problem-specific) architectures was addressed by resorting to genetic optimization. In this way, a total amount of 15 architectures were optimized, of which 6 were single-headed (three for each type of map), shown in Table 8 and Table 9 double-headed, as shown in Table 9. The performance results reported in Figure 12 (see the magnification in the upper left corner) confirm the superiority of the genetically optimized architectures over the pretrained ones. The drop in performance found with the transfer learning method can be explained by the fact that feature distributions across the two domains, source and target, were very different from each other. The pretrained models (Table 1), in fact, were pretrained using mostly “natural” images, while the images proposed in this study were obtained by mapping 3D point clouds in order to represent depths and normal vectors to the punch tool surface, resulting in “artificial” images with false colors.

Among the genetically optimized architectures, the single-headed models performed better than double-headed ones, with a slight predominance of models trained and tested on depth maps over those evaluated with normal maps. These results indicate that the use of two-headed models is not beneficial, and it is probably explained by poor correlations between features extracted from the two different types of maps, depth and normal, in representing deformation-induced degradation.

The results achieved in this study are in line with the state of the art in the literature. In particular, regarding CNN-based studies with image datasets; one of the best results presented in the literature was reported by Wu et al. [34]. In their study, the authors reported an average MAPE of 0.0476, which, however, was obtained with a very large data set consisting of 8400 images, while the TT was between 1.8 and 32.6 h (the authors have not provided specifications of used computing system). On the other hand, in the case of small data sets, one of the best results reported in the literature is that of Marei et al. [35] who achieved an average RMSE of 0.1654 with the Resnet18 pretrained model on a data set of 327 images, requiring 3358.4 s for fine-tuning on an NVIDIA GPU with 8GB Ram. However, it should be kept in mind that images of deteriorating components or parts of them (i.e., real world images, often obtained under a microscope and by stopping the machining system) were used in those studies. Therefore, the adaptation (or transfer learning) of pre-existing (or pretrained) CNN models was feasible, considering the similarity of feature distributions between domains. In the case, instead, of data sets consisting of time-series sensor data, Mo et al. [39] reported an average RMSE of 11.28 with the NASA C-MAPSS data set. A comparison between the results achieved in this study and the best results reported in the related literature is provided in Table 10.

The proposed system has been conceived to be versatile, working in a completely automatic way. There is no need to disassemble the punch tool or stop the punch machine to capture scans. The used 3D sensor, attached to the punching machine, scans at regular intervals of pressing stops. Furthermore, it is important to note that both depths, normal maps and longitudinal profiles, allow the punch tool RUL to be estimated in a single shot, i.e., a single profile or map accounts for all deformations that have occurred up to that moment. This allows the processing of long sequences of profiles or maps to be avoided, reducing computational load and network architecture complexity.

Although the optimization process takes a long time, it only needs to be performed once for the type of punch tool used. In this study, three different punch tools were tested, characterized by different deformation modes, using the same network architectures. An aspect that deserves further investigation concerns the verification of whether the proposed architectures are also valid for predicting the RUL of other systems than the studied punch tool, but whose degradation still depends on the work surface deformation.

## 6. Conclusions

In this study, a DNN-based RUL prediction framework for a punch tool, whose deterioration is due to surface deformation, was investigated. The RUL prediction is part of PHM maintenance strategies, in which the continuous monitoring of equipment health conditions allows the degradation state to be predicted, improving strategic maintenance decisions and thus avoiding late or too premature corrective actions. In the case of deformation-related degradations represented by DNVMs, the findings of this research show that the development of ad-hoc DNN models, driven by genetic optimization, provides better performance than pretrained models, indicating that the use of “transfer learning” is not always the best route.

The main results achieved are threefold, as indicated below. Firstly, the surface deformation of the punch tool was represented through the definition of DNVMs, obtained from point clouds of 3D scans. Secondly, the RUL prediction was estimated considering the main pretrained models, obtaining lower or slightly higher performance than SVR-based classic ML, due to the different distribution of features between the transfer learning domains. Thirdly, genetically optimized architectures based on a variable number of CNN blocks, both single- and double-headed, were generated, achieving superior performance to pretrained models and in line with the state of the art in the literature. Furthermore, the advantage of the proposed solution lies in its non-invasiveness and continuous operation. In fact, the monitoring is carried out by means of a 3D scan sensor placed outside the machinery, whose high degree of precision allows point clouds to be captured without stopping the punching process.

Ongoing and future research focuses on experimentation of DNVMs in combination with genetically optimized DNN architectures for the RUL prediction of other systems whose deterioration depends on surface deformations. More specifically, mechanical systems having geometry and material different from those of the punch tool will be the object of future investigations. This will allow verification of the degree of dependence of the DNN architecture on the characteristics of the mechanical system subject to degradation.

## Figures and Tables

**Table 1:** Pretrained networks evaluated for transfer learning.

Network	Input Size	$\mathrm{Parameters}\;(10^{6})$
Squeezenet [50]	227 × 227 × 3	1.2
Googlenet [51]	224 × 224 × 3	7.0
Inceptionv3 [52]	299 × 299 × 3	23.9
Densenet201 [53]	224 × 224 × 3	20.0
Mobilenetv2 [54]	224 × 224 × 3	3.5
Resnet18 [55]	224 × 224 × 3	11.7
Resnet50 [55]	224 × 224 × 3	25.6s×s
Resnet101 [55]	224 × 224 × 3	44.6
Xception [56]	299 × 299 × 3	22.9
Inceptionresnetv2 [57]	299 × 299 × 3	55.9
Shufflenet [58]	224 × 224 × 3	1.4
Nasnetmobile [59]	224 × 224 × 3	5.3
Nasnetlarge [59]	331 × 331 × 3	88.9
Darknet19 [60]	256 × 256 × 3	20.8
Darknet53 [60]	256 × 256 × 3	41.6
Efficientnetb0 [61]	224 × 224 × 3	5.3
Alexnet [62]	227 × 227 × 3	61.0
Vgg16 [63]	224 × 224 × 3	138.0
Vgg19 [63]	224 × 224 × 3	144.0

**Table 2:** Lower and upper bounds of all optimization variables.

Variable	$M_{i}$	Lower Bound	Upper Bound
Filter size $\left(x_{1},y_{1},y_{4}\right)$	4	1	2002
“	5	1	4004
“	6	1	6006
Number of filters $\left(x_{2},y_{2},y_{5}\right)$	4	1	35
“	5	1	126
“	6	1	462
Pooling positions $\left(x_{3},y_{3},y_{6}\right)$	4	1	16
“	5	1	32
“	6	1	64
Initial learning rate $\left(x_{4},y_{7}\right)$	4, 5, 6	$10^{-4}$	0.1
Momentum $\left(x_{5},y_{8}\right)$	4, 5, 6	0	1
Dropout probability $\left(y_{9},y_{10}\right)$	4, 5, 6	0	1

“ indicates a field identical to the one above.

**Table 3:** Kernel functions evaluated in this study.

Kernel	$G\left(x,y\right)$
Linear	〈x·y〉
Gaussian	$e-{∥x-y∥}^{2}$
Polynomial	${\left(1+\langle x\cdot y\rangle \right)}^{q}$ with $q\in \mathbb{N}\backslash \left\{0,1\right\}$

**Table 4:** RUL prediction performance obtained with genetically optimized networks tested on both depth and normal maps.

Model No.	Model Name	MAPE	RMSE	SF	TT (Sec)
1	go4normal	0.063	0.036	0.301	63.793
2	go5normal	0.065	0.037	0.313	18.351
3	go6normal	0.083	0.035	0.282	16.928
4	go4depth	0.102	0.063	0.513	13.647
5	go5depth	0.083	0.049	0.393	18.603
6	go6depth	0.058	0.036	0.312	13.410
7	go4+4	0.097	0.039	0.349	31.233
8	go4+5	0.141	0.057	0.493	125.250
9	go4+6	0.154	0.064	0.569	33.631
10	go5+4	0.123	0.048	0.427	96.948
11	go5+5	0.162	0.062	0.545	58.817
12	go5+6	0.129	0.053	0.469	58.204
13	go6+4	0.158	0.067	0.593	37.651
14	go6+5	0.237	0.082	0.762	196.594
15	go6+6	0.251	0.085	0.812	35.540

**Table 5:** RUL prediction performance obtained with pretrained networks tested on normal maps.

Model No.	Model Name	MAPE	RMSE	SF	FTT (Sec)
16	squeezenet	2.080	0.363	4.044	32.875
17	googlenet	0.452	0.112	1.077	18.947
18	inceptionv3	3.402	0.367	3.839	121.734
19	densenet201	1.802	0.559	5.453	758.300
20	mobilenetv2	2.692	0.608	6.471	94.666
21	resnet18	3.053	0.638	6.719	48.073
22	resnet50	1.854	0.426	4.086	140.182
23	resnet101	1.193	0.432	4.095	270.903
24	xception	1.825	0.365	1.550	833.500
25	inceptionresnetv2	2.691	0.555	5.365	755.150
26	shufflenet	2.673	0.905	9.534	73.828
27	nasnetmobile	1.597	0.412	4.276	352.860
28	darknet19	1.623	0.340	1.332	108.202
29	darknet53	1.384	0.485	1.870	565.150
30	efficientnetb0	1.408	0.338	3.306	195.668
31	alexnet	1.048	0.160	1.482	37.516
32	vgg16	0.615	0.311	1.177	310.489
33	vgg19	0.698	0.484	1.821	405.430

**Table 6:** RUL prediction performance obtained with pretrained networks tested on depth maps.

Model No.	Model Name	MAPE	RMSE	SF	FTT (Sec)
34	squeezenet	3.789	0.321	3.175	36.501
35	googlenet	0.416	0.114	1.142	56.326
36	inceptionv3	2.200	0.312	3.067	267.350
37	densenet201	1.657	0.527	5.387	785.250
38	mobilenetv2	2.108	0.452	4.768	104.493
39	resnet18	1.602	0.448	4.576	48.366
40	resnet50	1.181	0.312	2.963	147.724
41	resnet101	1.838	0.633	7.828	283.090
42	xception	1.790	0.409	1.725	915.500
43	inceptionresnetv2	1.530	0.266	2.519	745.000
44	shufflenet	2.062	0.694	7.401	74.328
45	nasnetmobile	1.309	0.324	3.329	345.805
46	darknet19	9.471	3.291	14.630	126.012
47	darknet53	0.878	0.423	1.557	580.700
48	efficientnetb0	0.870	0.274	2.761	183.381
49	alexnet	0.822	0.311	2.921	35.722
50	vgg16	1.095	0.616	2.476	312.283
51	vgg19	0.648	0.452	1.683	409.718

**Table 7:** RUL prediction performance obtained with SVR algorithms tested on surface profiles.

Model No.	Model Name	MAPE	RMSE	SF	TT (Sec)
52	linear	1.073	0.135	1.444	4.667
53	gaussian	1.190	0.182	1.673	4.117
54	polynomial3	1.107	0.165	1.502	5.053
55	polynomial4	0.909	0.124	1.180	5.726
56	polynomial5	0.862	0.113	1.134	5.734
57	polynomial6	0.857	0.120	1.179	5.807

**Table 8:** Network configuration of genetically optimized single-head DNN architectures.

Model No.	Model Name	Network Architecture	$\mathrm{Parameters}\;(10^{6})$
1	go4normal	**S** = (29,25,19,17)	1.39
		**N** = (32,32,32,32)	
		**P** = (0,0,0,0)	
		**ILR** = 0.0059	
		**M** = 0.8634	
2	go5normal	**S** = (5,7,19,23,29)	4.95
		**N** = (32,32,32,64,64)	
		**P** = (1,0,0,0,0)	
		**ILR** = 0.0068	
		**M** = 0.8049	
3	go6normal	**S** = (29,25,23,19,11,7)	1.72
		**N** = (16,32,32,32,64,64)	
		**P** = (1,1,1,1,0,0)	
		**ILR** = 0.0047	
		M = 0.7572	
4	go4depth	**S** = (29,23,21,15)	0.347
		**N** = (16,16,16,16)	
		**P** = (1,1,1,1)	
		**ILR** = 0.0074	
		**M** = 0.6636	
5	go5depth	**S** = (29,27,15,11,3)	1.19
		**N** = (32,32,32,32,32)	
		**P** = (1,1,1,0,0)	
		**ILR** = 0.0054	
		**M** = 0.6893	
6	go6depth	**S** = (3,5,15,21,23,27)	7.93
		**N** = (32,64,64,64,64,64)	
		**P** = (1,0,0,0,0,0)	
		**ILR** = 0.0077	
		**M** = 0.7702	

**Table 9:** Network configuration of genetically optimized double-head DNN architectures.

Model No.	Model Name	Network Architecture	$\mathrm{Parameters}\;(10^{6})$
7	go4+4	**S1** = (5,15,21,29), **S2** = (29,27,25,17) **N1** = (16,16,16,32), **N2** = (16,16,16,16) **P1** = (1,1,1,1), **P2** = (1,1,1,1) d1 = 0.6791, d2 = 0.2646 ILR = 0.0086 M = 0.6499	1.06
8	go4+5	**S1** = (27,23,19,3), **S2** = (29,25,23,13,7) **N1** = (32,32,32,32), **N2** = (16,64,64,64,64) **P1** = (1,0,0,0), **P2** = (0,0,0,0,0) d1 = 0.2452, d2 = 0.2494 ILR = 0.0084 M = 0.5046	4.73
9	go4+6	**S1** = (5,23,27,29), **S2** = (27,25,23,17,13,9) **N1** = (16,32,32,32), **N2** = (16,16,16,32,64,64) **P1** = (1,0,0,0), **P2** = (0,0,0,0,0,0) d1 = 0.2659, d2 = 0.4718 ILR = 0.0069 M = 0.6599	7.76
10	go5+4	**S1** = (27,25,23,21,17), **S2** = (29,25,11,5) **N1** = (16,32,32,32,64), **N2** = (32,32,32,32) **P1** = (0,0,0,0,0), **P2** = (0,0,0,0) d1 = 0.598, d1 = 0.282 ILR = 0.008 M = 0.804	2.81
11	go5+5	**S1** = (25,21,19,15,7), **S2** = (29,21,15,13,3) **N1** = (64,64,64,64,64), **N2** = (16,32,32,64,64) **P1** = (1,1,1,0,0), **P2** = (0,0,0,0,0) d1 = 0.1296, d2 = 0.1075 ILR = 0.0089 M = 0.4570	5.41
12	go5+6	**S1** = (9,11,13,25,27), **S2** = (7,11,17,19,21,27) **N1** = (16,16,32,64,64), **N2** = (16,32,64,64,64,64) **P1** = (1,0,0,0,0), **P2** = (0,0,0,0,0,0) d1 = 0.425, d1 = 0.355 ILR = 0.006 M = 0.464	11.32
13	go6+4	**S1** = (5,15,19,21,23,29), **S2** = (11,17,19,25) **N1** = (16,16,32,32,32,32), **N2** = (16,16,32,32) **P1** = (1,0,0,0,0,0), **P2** = (1,1,0,0) d1 = 0.7385, d2 = 0.5250 ILR = 0.0039 M = 0.1515	3.00
14	go6+5	**S1** = (29,27,19,17,13,5), **S2** = (7,17,21,27,29) **N1** = (64,64,64,64,64,64), **N2** = (16,32,32,32,32) **P1** = (0,0,0,0,0,0), **P2** = (1,0,0,0,0) d1 = 0.8581, d2 = 0.5764 ILR = 0.0074 M = 0.5507	8.82
15	go6+6	**S1** = (3,13,15,21,25,29), **S2** = (3,11,13,17,21,25) **N1** = (16,16,16,32,64,64), **N2** = (16,16,64,64,64,64) **P1** = (1,0,0,0,0,0), **P2** = (1,0,0,0,0,0) d1 = 0.7867, d2 = 0.6495 ILR = 0.0046 M = 0.6290	10.81

**Table 10:** Comparison between related studies and this study.

Study	Dataset	Degradation	Approach	MAPE	RMSE	TT/FTT
Wu et al. [32]	8400 camera images	Tool wear	Transfer learning	0.0476	-	2 ÷ 333 h
Marei et al. [33]	327 camera images	Tool wear	Transfer learning	-	0.1654	3358 s
Mo et al. [37]	21 time series (engine sensors)	Turbofan engine	Genetically optimized	-	11.28	-
Ren et al. [19]	15 time series (vibration sensor)	Bearing wear	Manually engineered	-	0.2	-
This study	2014 scans	Punch tool	Transfer learning	0.416	0.112	19 ÷ 916 s
This study	2014 scans	Punch tool	Genetically optimized	0.058	0.035	13 ÷ 125 s

## Data Availability

Not applicable.
